# Is It True?

**Published:** 1874-11

**Authors:** 


### IS IT TRUE ?

A GENTLEMAN who has been studying up the subject, and has examined many dogs and cats in France, England, and America, says he has discovered that every spotted dog has the end of his tail white, and every spotted cat the end of the tail black. Is it true ? Look at your pussy and your dog, and at the cats and dogs which you meet.

